# Synthesis of Oxylipin Mimics and Their Antifungal Activity against the Citrus Postharvest Pathogens

**DOI:** 10.3390/molecules21020254

**Published:** 2016-02-22

**Authors:** Jimei Ma, Yupeng Li, Hangwei Chen, Zhen Zeng, Zi-Long Li, Hong Jiang

**Affiliations:** Department of Chemistry, College of Sciences, Huazhong Agricultural University, Wuhan 430070, China; majimei@mail.hzau.edu.cn (J.M.); liyupeng@webmail.hzau.edu.cn (Y.L.); chw234234@hotmail.com (H.C.); zengzhen@mail.hzau.edu.cn (Z.Z.); lizilong@mail.hzau.edu.cn (Z.-L.L.)

**Keywords:** oxylipin, synthesis, antifungal activity, citrus, pathogen

## Abstract

Nine oxylipin mimics were designed and synthesized starting from d-mannose. Their antifungal activity against three citrus postharvest pathogens was evaluated by spore germination assay. The results indicated that all the compounds significantly inhibited the growth of *Penicillium digitatum*, *Penicillium italicum* and *Aspergillus niger*. The compound (3*Z*,6*Z*,8*S*,9*R*,10*R*)-octadeca-3,6-diene-8,9,10-triol (**3**) exhibited excellent inhibitory effect on both *Penicillium digitatum* (IC_50_ = 34 ppm) and *Penicillium italicum* (IC_50_ = 94 ppm). Their *in vivo* antifungal activities against citrus postharvest blue mold were tested with fruit inoculated with the pathogen *Penicillium italicum*. The compound (3*R*,4*S*)-methyl 3,4-dihydroxy-5-octyltetrahydrofuran-2-carboxylate (**9**) demonstrated significant efficacy by reducing the disease severity to 60%. The antifungal mechanism of these oxylipin mimics was postulated in which both inhibition of pathogenic mycelium and stimuli of the host oxylipin-mediated defense response played important roles.

## 1. Introduction

The biologically active compounds oxidized from polyunsaturated fatty acids in aerobic organisms are collectively termed oxylipins, which play an important role in a variety of functions including growth, aging, development, and defense responses to environmental stimuli [1,2,3,4]. Generally, oxylipins are enzymatically or spontaneously synthesized *de novo* in response to mechanical injury, pathogen attack and other environmental inputs [5,6,7]. The chemical structures of oxylipins are diverse including hydroxyl, aldehyde, epoxy, ketol and divinyl-ether derivatives. It has been reported that quite a few oxylipins and their metabolisms play important roles in defense against microbial pathogens [8,9]. For example, linolenic acid and linoleic acid exhibited antifungal activity against *Rhizoctonia solani* and *Pythium ultimum* [10]. The compound 12-oxo-10,15(*Z*)-phytodienoic acid strongly inhibited mycelial growth and spore germination of eukaryotic microbes [8]. Jasmonic acid could suppress the reproductive development and secondary metabolism of *Aspergillus* specie [11,12]. Free fatty acid methyl ester fractions isolated from *Linum usitatissimum* L. seeds exhibited antifungal activity against toxigenic *Apsergillus* [13,14].

**Scheme 1:**
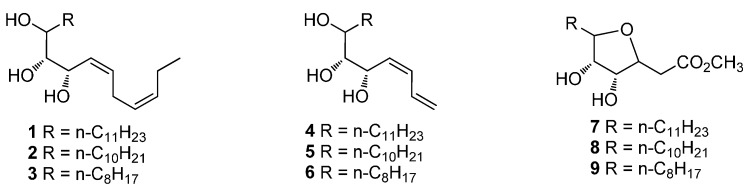
The structures of designed oxylipin mimics.

Citrus fruit is one of the most abundant agricultural economic crops in the world due to its taste and nutritive properties. However, decay during postharvest storage always brings significant loss in the economy [15]. The most common culprits are green and blue mold [16,17,18,19]. At present, the application of germicides is a principal method to control postharvest decay. The safety and endurance of these compounds are mainly considered, besides the preservative effect. Oxylipin, which is a natural plant metabolite and generally low in toxicity, was reported to show notable antifungal activity. In this paper, a series of oxylipin mimics were designed and synthesized (Scheme 1). Their antifungal effects on *P. digitatum*, *P. italicum* and *Aspergillus niger* in Petri dishes were also investigated.

## 2. Results and Discussion

### 2.1. Synthetic Chemistry

In this work, we designed a few oxylipin mimics which were mainly unsaturated alkane with a multi-hydroxyl group forming an aliphatic carbon chain ranging from 15 to 21. Furthermore, a few mimics with a tetrahydrofuran ring were also synthesized to investigate the influence of different structures on antifungal activities [2,20].

**Scheme 2:**
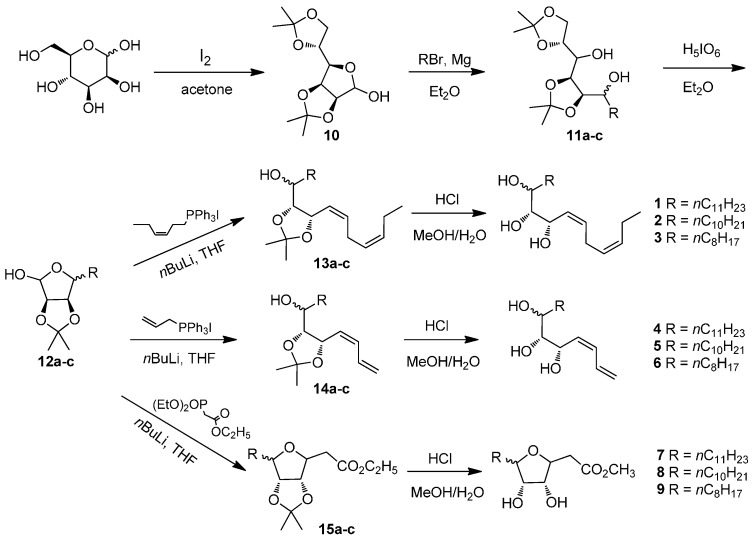
The synthetic route to compounds **1**–**9**. **11a**, **12a**, **13a**, **14a**, **15a**: R = *n*C_11_H_23_; **11b**, **12b**, **13b**, **14b**, **15b**: R = *n*C_10_H_21_; **11c**, **12c**, **13c**, **14c**, **15c**: R = *n*C_8_H_17_.

The compounds **1**–**9** were synthesized from d-mannose, which provided inherent multi-hydroxyl groups. The synthetic route of compounds **1**–**9** was shown in Scheme 2. The compound 2,3:5,6-di-*O*-isopropylidene-d-mannose (**10**), prepared according to the literature [21], was reacted with the corresponding Grignard reagent to give compound **11**. Oxidation of compound **11** with periodic acid [22] afforded compound **12**. The Wittig/Horner-Wadsworth-Emmons reaction of compound **12** and subsequent deprotection resulted in the desired oxylipin mimics **1**–**9** [23,24]. All the synthesized products were characterized by ^1^H-NMR and HRMS.

### 2.2. Evaluation of in Vitro Antifungal Activity

All the synthesized compounds were screened to investigate their antifungal activities against *P. digitatum*, *P. italicum* and *A. niger* on Petri dishes using the agar dilution method. Linolenic acid was reported to inhibit a few fungi [10,25] and was thus used as the reference compound. IC_50_, the concentration inhibiting 50% of fungal activity, was calculated and shown in Table 1. All the tested compounds showed significant antifungal activities against *P. digitatumi* and diverse activities against *P. italicum* and *A. niger*. Compound **2** strongly reduced the conidia germination of *P. digitatumi* with the IC_50_ value of 23 ppm. Compound **3** demonstrated substantial antifungal activity against *P. italicum* (IC_50_ = 94 ppm). Compound **9** inhibited the growth of *A. niger* with the IC_50_ value of 202 ppm.

**Table 1:** IC_50_ values of the tested compounds against three citrus postharvest pathogens.

Compound	IC_50_ for *Penicillium digitatumi* (ppm)	IC_50_ for *Penicillium italicum* (ppm)	IC_50_ for *Aspergillus niger* (ppm)
**1**	35.54 ± 1.75	133.12 ± 2.27	256.22 ± 18.93
**2**	22.73 ± 3.43	114.01 ± 7.63	385.56 ± 12.74
**3**	33.64 ± 1.85	94.41 ± 7.62	311.04 ± 5.71
**4**	47.59 ± 0.13	156.97 ± 10.13	243.64 ± 11.31
**5**	31.45 ± 3.26	191.37 ± 8.14	301.01 ± 3.54
**6**	28.43 ± 0.82	182.33 ± 13.50	263.81 ± 8.84
**7**	201.65 ± 6.48	272.83 ± 18.73	257.99 ± 9.32
**8**	59.73 ± 4.31	208.85 ± 12.28	333.15 ± 13.62
**9**	123.69 ± 8.79	212.60 ± 11.08	202.40 ± 2.99
Linolenic acid	57.96 ± 2.16	234.21 ± 10.43	272.14 ± 5.47

Compounds **1**–**9** showed antifungal activities against three pathogens. It is reasonable to speculate that compounds possessing many hydroxylic groups are able to interact with fungal cytoderm composed mainly from chitin. This results in increased cytoderm permeability [26]. If considering structural features, triols **1**–**6** containing long aliphatic chains showed better antifungal activities than compounds **7**–**9**, which were characterized by cyclic structure and a lower number of hydroxyl groups. It seems that the length of the chain for similar structures did not show an apparent difference. Overall, oxylipin mimic treatment could result in the inhibition of germination or spore death.

### 2.3. Evaluation of in Vivo Antifungal Activity

To further explore the antifungal activities of these oxylipin mimics, the test to control citrus postharvest blue mold on fruit inoculated with the pathogens was conducted. Results summarized in Table 2 showed that all the compounds reduced the severity of blue mold to some extent as compared to the control after 10 days. The most effective, compound **3**, in spore germination assay simply reduced the disease severity to 82.5%. However, compound **9** exhibited the best antifungal activity *in vivo*. Compound **9** reduced the disease severity to 60%, which is more effective than that of linolenic acid (65%).

**Table 2:** The disease index after 10 days on citrus treated with tested compounds.

Compound	Disease Index (%)
Control	97.5
**1**	80.0
**2**	75.0
**3**	82.5
**4**	90.0
**5**	77.5
**6**	65.0
**7**	62.5
**8**	65.0
**9**	60.0
Linolenic acid	65.0

It is noteworthy that the antifungal activities of the tested compounds against *P. italicum* on citrus and *in vitro* Petri dish are inconsistent. Compounds **7**–**9**, which displayed moderate inhibitory effect on *P. italicum* in a Petri dish, showed better a inhibitory effect *in vivo* than those compounds without an ester group. It is possible that the ester group could be a contributing factor which could be converted to acid by hydrolase. It is well known that the antifungal activity of oxylipin is not simply due to the inhibition of the growth of mycelia or killing fungi spores directly. Oxylipin may act as a signal-transmitting substance to activate the immunomodulation properties of the host to induce a systemic defense response [6]. The reactive oxygen species [27] and related enzymes [1,28] such as lipoxygenase, α-dioxygenase and allene oxide synthase could also be influenced by oxylipin. Whether the compounds in this work demonstrate a significant effect through similar mechanisms is worth investigating in the future.

## 3. Experimental Section

### 3.1. General

All reagents were commercially available and used without further purification. Solvents were dried prior to use. All the reactions were performed under argon atmosphere. The ^1^H-NMR spectra were collected at room temperature on 400 MHz Bruker AM spectrometers (Bruker Corporation, Madison, WI, USA). The residual solvent signals were taken as the reference (7.26 ppm for ^1^H-NMR and 77.0 ppm for ^13^C-NMR in CDCl_3_). Chemical shift (δ) is reported in ppm, coupling constants (*J*) are given in Hz. The following abbreviations classify the multiplicity: s = singlet, brs = broad singlet, d = doublet, t = triplet, m = multiplet or unresolved. HR-MS (ESI) spectra were recorded on Waters Q-Tof premier™ (Waters Corporation, Milford, MA, USA) and Bruker MicroTOF II mass spectrometer (Bruker Corporation). The ^1^H-NMR and HRMS spectra were attached in the Supplementary Materials.

### 3.2. Synthesis of Compounds ***1**–**9***

*(2R,4S,5R)-1,2:4,5-Diisopropylideneundecyl-1,2,3,4,5,6-hexaol* (**11a**). To a suspension of magnesium turnings (3.65 g, 0.15 mol) in 100 mL of anhydrous ether, 1-bromoundecane (4.7 g, 0.02 mol) was added. The reaction was initiated by addition of 0.1 mL dibromoethane. Then 1-bromoundecane (18.8 g, 0.08 mol) was added dropwise to keep gently refluxing. After 3 h, the mixture was cooled to room temperature and then −10 °C, which was followed by addition of compound **10** (2.6 g, 0.01 mol). After completion of addition, the mixture was warmed to room temperature and stirred for 2 h. Ice water was added to quench the reaction and the resulting suspension was filtered through Celite. The filtrate was concentrated and purified by column chromatography on silica gel (ethyl acetate/petroleum ether = 1:5) to give compound **11a** as a colorless oil (3.54 g, 85%). ^1^H-NMR (400 MHz, CDCl_3_) δ: 0.87 (t, *J* = 6.8 Hz, 3H, CH_3_), 1.23–1.53 (m containing 4 s, 32H, 4CH_3_, 10CH_2_), 3.59–3.62 (m, 1H, CH), 3.77–3.88 (m, 1H, CH), 3.97–4.14 (m, 4H, 2CH, CH_2_), 4.34–4.41 (m, 1H, CH); HRMS (ESI) *m*/*z* 439.3036 (calcd. for C_24_H_44_O_6_Na [M + Na]^+^, 439.3036).

*(3R,4S)-3,4-Isopropylidene-5-undecyltetrahydrofuran-2,3,4-triol* (**12a**). A mixture of periodic acid (3.84 g, 16.95 mmol) and compound **11a** (2.35 g, 5.65 mmol) in 200 mL of anhydrous ether was stirred for 4 h at room temperature. The suspension was filtered to remove the extra periodic acid. The filtrate was washed with saturated NaHCO_3_ solution to neutral and then washed with brine. The organic phase was dried over Na_2_SO_4_, filtered and concentrated. The residue was purified by column chromatography on silica gel (ethyl acetate/petroleum ether = 1:10) to give compound **12a** as a colorless oil (1.55 g, 87%). ^1^H-NMR (400 MHz, CDCl_3_) δ: 0.86 (t, *J* = 6.8 Hz, 3H, CH_3_), 1.24–1.46 (m containing 2 s, 24H, 2CH_3_, 9CH_2_), 1.68 (dd, *J* = 14.7, 7.2 Hz, 2H, CH_2_), 3.31(s, 1H, OH), 4.10 (dt, *J* = 6.8, 3.6 Hz, 1H, CH), 4.56–4.57 (m, 1H, CH), 4.60–4.64 (m, 1H, CH), 5.32 (d, *J* = 1.6 Hz, 1H, CH); HRMS (ESI) *m*/*z* 337.2355 (calcd. for C_18_H_34_O_6_Na [M + Na]^+^, 337.2354).

*(3Z,6Z,8S,9R)-8,9-Isopropylidene-heneicosa-3, 6-diene-8, 9, 10-triol* (**13a**). *n*-Butyl lithium (9.88 mL, 1.6 M) was added dropwise to a solution of (*Z*)-hex-3-ene triphenylphosphonium iodine (5.81 g, 12.3 mmol) in 70 mL of THF at −30 °C. After 0.5 h, a solution of compound **12a** (1.10 g, 3.5 mmol) in 5 mL of THF was added to the mixture, which was then allowed to warm to 0 °C. The reaction was continued for another 6 h. The resulting mixture was filtered. The filtrate was dried over Na_2_SO_4_, filtered and concentrated. The residue was purified by column chromatography on silica gel (ethyl acetate/petroleum ether = 1:20) to give compound **13a** as a colorless oil (0.65 g, 49%). ^1^H-NMR (400 MHz, CDCl_3_) δ: 0.88 (t, *J* = 6.8 Hz, 3H, CH_3_), 0.97 (t, *J* = 7.4 Hz, 3H, CH_3_), 1.26–1.51 (m containing 2 s, 26H, 2CH_3_, 10CH_2_), 2.02–2.10 (m, 2H, CH_2_), 2.80–2.86 (m, 2H, CH_2_), 3.56–3.59 (m 1H, CH), 3.97–4.00 (m, 1H, CH), 4.98 (dd, *J* = 8.6, 6.8 Hz, 1H, CH), 5.24–5.74 (m, 4H, 2CH=CH); HRMS (ESI) *m*/*z* 403.3191 (calcd. for C_24_H_44_O_3_Na [M + Na]^+^, 403.3188).

*(3Z,6Z,8S,9R,10R)-Henicosa-3,6-diene-8,9,10-triol* (**1**). Compound **13a** (0.46 g, 1.2 mmol) was added to a solution of concentrated HCl (11 mL) in water/methanol (75 mL, *v*/*v* = 1:9). The mixture was stirred for 3 h at room temperature and then neutralized with NaOH solution. The resulting mixture was concentrated to remove methanol. The residue was diluted with EtOAc and washed with water. The organic phase was dried over Na_2_SO_4_, filtered and concentrated. The residue was purified by column chromatography on silica gel (ethyl acetate/petroleum ether = 1:2) to give compound **1** as a colorless oil (0.32 g, 78%). ^1^H-NMR (400 MHz, CDCl_3_) δ: 0.87 (t, *J* = 6.8 Hz, 3H, CH_3_), 0.97 (t, *J* = 7.5 Hz, 3H, CH_3_), 1.25–1.51 (m, 20H, 10CH_2_), 2.02–2.08 (m, 2H, CH_2_), 2.15 (brs, 3H, 3OH), 2.79–2.89 (m, 2H, CH_2_), 3.28–3.48 (m, 1H, CH), 3.64–3.85 (m, 1H, CH), 4.22–4.67 (m, 1H, CH), 5.27–5.83 (m, 4H, CH=CH); HRMS (ESI) *m*/*z* 363.2875 (calcd. for C_21_H_40_O_3_Na [M + Na]^+^, 363.2875).

*(3Z,6Z,8S,9R,10R)-Icosa-3,6-diene-8,9,10-triol* (**2**). To a suspension of magnesium turnings (4.86 g, 0.2 mol) in 200 mL of anhydrous ether, 1-bromodecane (8.85 g, 0.04 mol) was added. The reaction was initiated by addition of 0.1 mL dibromoethane. Then 1-bromodecane (35.39 g, 0.16 mol) was added dropwise to keep gently refluxing. After 3 h, the mixture was cooled to room temperature and then −10 °C, which was followed by addition of compound **10** (5.21 g, 0.02 mol). After completion of addition, the mixture was warmed to room temperature and stirred for 2 h. Ice water was added to quench the reaction and the resulting suspension was filtered through Celite. The filtrate was concentrated and purified by column chromatography on silica gel (ethyl acetate/petroleum ether = 1:5) to give compound **11b** as a colorless oil (7.08 g, 88%). A mixture of periodic acid (11.97 g, 52.5 mmol) and compound **11b** (7.04 g, 17.5 mmol) in 250 mL of anhydrous ether was stirred for 4 h at room temperature. The suspension was filtered to remove the extra periodic acid. The filtrate was washed with saturated NaHCO_3_ solution to neutral and then washed with brine. The organic phase was dried over Na_2_SO_4_, filtered and concentrated. The residue was purified by column chromatography on silica gel (ethyl acetate/petroleum ether = 1:10) to give compound **12b** as a colorless oil (4.16 g, 79%). *n*-Butyl lithium (11.8 mL, 1.6 M) was added dropwise to a solution of (*Z*)-hex-3-ene triphenylphosphonium iodine (7.44 g, 15.75 mmol) in 80 mL of THF at −30 °C. After 0.5 h, a solution of compound **12b** (1.35 g, 4.5 mmol) in 5 mL of THF was added to the mixture, which was then allowed to warm to 0 °C. The reaction was continued for another 6 h. The resulting mixture was filtered. The filtrate was dried over Na_2_SO_4_, filtered and concentrated. The residue was purified by column chromatography on silica gel (ethyl acetate/petroleum ether = 1:20) to give compound **13b** as a colorless oil (0.76 g, 46%). Compound **13b** (0.75 g, 2.05 mmol) was added to a solution of concentrated HCl (8.5 mL) in water/methanol (100 mL, *v*/*v* = 1:9). The mixture was stirred for 3 h at room temperature and then neutralized with NaOH solution. The resulting mixture was concentrated to remove methanol. The residue was diluted with EtOAc and washed with water. The organic phase was dried over Na_2_SO_4_, filtered and concentrated. The residue was purified by column chromatography on silica gel (ethyl acetate/petroleum ether = 1:2) to give compound **2** as a colorless oil (0.48 g, 72%). ^1^H-NMR (400 MHz, CDCl_3_) δ: 0.88 (t, *J* = 6.4 Hz, 3H, CH_3_), 0.97 (t, *J* = 7.5 Hz, 3H, CH_3_), 1.24–1.73 (m, 18H, 9CH_2_), 2.00–2.10 (m containing brs, 3H, CH_2_, OH), 2.44 (brs, 1H, OH), 2.63 (brs, 1H, OH), 2.77–2.91 (m, 2H, CH_2_), 3.39–3.46 (m, 1H, CH), 3.66–3.83 (m, 1H, CH), 4.22–4.30 (m, 0.5H, CH), 4.64–4.66 (m, 0.5H, CH), 5.28–5.83 (m, 4H, 2CH=CH); HRMS (ESI) *m*/*z* 349.2713 (calcd. for C_20_H_38_O_3_Na [M + Na]^+^, 349.2706).

*(3Z,6Z,8S,9R,10R)-Octadeca-3,6-diene-8,9,10-triol* (**3**). To a suspension of magnesium turnings (4.86 g, 0.2 mol) in 200 mL of anhydrous ether, 1-bromoctane (7.72 g, 0.04 mol) was added. The reaction was initiated by addition of 0.1 mL dibromoethane. Then 1-bromodecane (30.9 g, 0.16 mol) was added dropwise to keep gently refluxing. After 3 h, the mixture was cooled to room temperature and then −10 °C, which was followed by addition of compound **10** (5.2 g, 0.02 mol). After completion of addition, the mixture was warmed to room temperature and stirred for 2 h. Ice water was added to quench the reaction and the resulting suspension was filtered through Celite. The filtrate was concentrated and purified by column chromatography on silica gel (ethyl acetate/petroleum ether = 1:5) to give compound **11c** as a colorless oil (5.91 g, 79%). A mixture of periodic acid (10.77 g, 47.25 mmol) and compound **11c** (5.90 g, 15.75 mmol) in 250 mL of anhydrous ether was stirred for 4 h at room temperature. The suspension was filtered to remove the extra periodic acid. The filtrate was washed with saturated NaHCO_3_ solution to neutral and then washed with brine. The organic phase was dried over Na_2_SO_4_, filtered and concentrated. The residue was purified by column chromatography on silica gel (ethyl acetate/petroleum ether = 1:10) to give compound **12c** as a colorless oil (3.36 g, 78%). *n*-Butyl lithium (10.5 mL, 1.6 M) was added dropwise to a solution of (*Z*)-hex-3-ene triphenylphosphonium iodine (6.61 g, 14 mmol) in 80 mL of THF at −30 °C. After 0.5 h, a solution of compound **12c** (1.1 g, 4 mmol) in 5 mL of THF was added to the mixture, which was then allowed to warm to 0 °C. The reaction was continued for another 6 h. The resulting mixture was filtered. The filtrate was dried over Na_2_SO_4_, filtered and concentrated. The residue was purified by column chromatography on silica gel (ethyl acetate/petroleum ether = 1:20) to give compound **13c** as a colorless oil (0.48 g, 36%). Compound **13c** (0.47 g, 1.4 mmol) was added to a solution of concentrated HCl (6 mL) in water/methanol (100 mL, *v*/*v* = 1:9). The mixture was stirred for 3 h at room temperature and then neutralized with NaOH solution. The resulting mixture was concentrated to remove methanol. The residue was diluted with EtOAc and washed with water. The organic phase was dried over Na_2_SO_4_, filtered and concentrated. The residue was purified by column chromatography on silica gel (ethyl acetate/petroleum ether = 1:2) to give compound **3** as a colorless oil (0.39 g, 75%). ^1^H-NMR (400 MHz, CDCl_3_) δ: 0.88 (t, *J* = 6.8 Hz, 3H, CH_3_), 0.96(t, *J* = 7.5 Hz, 3H, CH_3_), 1.26–1.72(m, 14H, CH_2_), 2.02–2.06(m, 2H, CH_2_), 2.20 (brs, 2H, 2OH), 2.60(brs, 1H, OH), 2.77–2.86 (m, 2H, CH_2_), 3.44–3.48 (m, 1H, CH), 3.65(s, 1H, CH), 4.23 (s, 1H, CH), 5.30–5.37 (m, 1H, =CH), 5.43–5.49(m, 1H, =CH), 5.58–5.64 (m, 1H, =CH), 5.77–5.84(m, 1H, =CH); HRMS (ESI) *m*/*z* 321.2405 (calcd. for C_18_H_34_O_3_Na [M + Na]^+^, 321.2400).

*(5S,6R,7R,Z)-Octadeca-1,3-diene-5,6,7-triol* (**4**). *n*-Butyl lithium (8.5 mL, 1.6 M) was added dropwise to a solution of allyl triphenylphosphonium bromide (4.02 g, 10.5 mmol) in 60 mL of THF at −10 °C. After 0.5 h, a solution of compound **12a** (0.94 g, 3.0 mmol) in 5 mL of THF was added to the mixture, which was then allowed to warm to 20 °C. The reaction was continued for another 6 h. The resulting mixture was filtered. The filtrate was dried over Na_2_SO_4_, filtered and concentrated. The residue was purified by column chromatography on silica gel (ethyl acetate/petroleum ether = 1:20) to give compound **14a** as a colorless oil (0.39 g, 40%). Compound **14a** (0.37 g, 1.1 mmol) was added to a solution of concentrated HCl (10 mL) in water/methanol (70 mL, *v*/*v* = 1:9). The mixture was stirred for 3 h at room temperature and then neutralized with NaOH solution. The resulting mixture was concentrated to remove methanol. The residue was diluted with EtOAc and washed with water. The organic phase was dried over Na_2_SO_4_, filtered and concentrated. The residue was purified by column chromatography on silica gel (ethyl acetate/petroleum ether = 1:3) to give compound **4** as a colorless oil (0.24 g, 74%). ^1^H-NMR (400 MHz, CDCl_3_) δ: 0.88 (t, *J* = 6.8 Hz, 3H, CH_3_), 1.01–1.73 (m, 20H, 10CH_2_), 3.34–3.37 (m, 1H, CH), 3.54–3.97 (m, 2H, 2CH), 5.14–5.34 (m, 2H, =CH_2_), 5.57–5.82 (m, 1H, =CH), 6.22–6.72 (m, 2H, 2CH=); HRMS (ESI) *m*/*z* 321.2404 (calcd. for C_18_H_34_O_3_Na [M + Na]^+^, 321.2400).

*(5S,6R,7R,Z)-Heptadeca-1,3-diene-5,6,7-triol* (**5**). *n*-Butyl lithium (11.8 mL, 1.6 M) was added dropwise to a solution of allyl triphenylphosphonium bromide (6.77 g, 15.75 mmol) in 80 mL of THF at −10 °C. After 0.5 h, a solution of compound **12b** (1.35 g, 4.5 mmol) in 5 mL of THF was added to the mixture, which was then allowed to warm to 20 °C. The reaction was continued for another 6 h. The resulting mixture was filtered. The filtrate was dried over Na_2_SO_4_, filtered and concentrated. The residue was purified by column chromatography on silica gel (ethyl acetate/petroleum ether = 1:20) to give compound **14b** as a colorless oil (0.65 g, 45%). Compound **14b** (0.65 g, 2 mmol) was added to a solution of concentrated HCl (8 mL) in water/methanol (50 mL, *v*/*v* = 1:9). The mixture was stirred for 3 h at room temperature and then neutralized with NaOH solution. The resulting mixture was concentrated to remove methanol. The residue was diluted with EtOAc and washed with water. The organic phase was dried over Na_2_SO_4_, filtered and concentrated. The residue was purified by column chromatography on silica gel (ethyl acetate/petroleum ether = 1:3) to give compound **5** as a colorless oil (0.38 g, 68%). ^1^H-NMR (400 MHz, CDCl_3_) δ: 0.87 (t, *J* = 6.8 Hz, 3H), 1.25–1.62 (m, 18H), 2.27–2.85 (brs, 1H, OH), 3.04–3.18 (brs, 1H, OH), 3.83–3.89 (m, 2H, 2CH), 3.99–4.00 (m, 1H, CH), 4.04 (s, 1H, OH), 4.74–4.89 (m, 2H, =CH_2_), 5.22–5.55 (m, 2H, 2=CH), 6.15–6.66 (m, 1H, =CH); HRMS (ESI) *m*/*z* 307.2250 (calcd. for C_17_H_32_O_3_Na [M + Na]^+^, 307.2244).

*(5S,6R,7R,Z)-Pentadeca-1,3-diene-5,6,7-triol* (**6**). *n*-Butyl lithium (10.5 mL, 1.6 M) was added dropwise to a solution of allyl triphenylphosphonium bromide (6.02 g, 14 mmol) in 80 mL of THF at −10 °C. After 0.5 h, a solution of compound **12c** (1.1 g, 4 mmol) in 5 mL of THF was added to the mixture, which was then allowed to warm to 20 °C. The reaction was continued for another 6 h. The resulting mixture was filtered. The filtrate was dried over Na_2_SO_4_, filtered and concentrated. The residue was purified by column chromatography on silica gel (ethyl acetate/petroleum ether = 1:20) to give compound **14c** as a colorless oil (0.49 g, 41%). Compound **14c** (0.48 g, 1.6 mmol) was added to a solution of concentrated HCl (6.7 mL) in water/methanol (40 mL, *v*/*v* = 1:9). The mixture was stirred for 3 h at room temperature and then neutralized with NaOH solution. The resulting mixture was concentrated to remove methanol. The residue was diluted with EtOAc and washed with water. The organic phase was dried over Na_2_SO_4_, filtered and concentrated. The residue was purified by column chromatography on silica gel (ethyl acetate/petroleum ether = 1:3) to give compound **6** as a colorless oil (0.31 g, 76%). ^1^H NMR (400 MHz, CDCl_3_) δ: 0.89 (t, *J* = 6.4 Hz, 3H, CH_3_), 1.25–1.59 (m, 14H, 7CH_2_), 2.98 (brs, 3H, 3OH), 3.33–3.36 (m, 0.5H, CH), 3.41–3.42 (m, 0.5H, CH), 3.50–3.53 (m, 0.5H, CH), 3.62–3.65 (m, 0.5H, CH), 3.77–3.86 (m, 0.5H, CH), 3.94–4.03 (m, 0.5H, CH), 4.31–4.78 (m, 1H, =CH_2_), 5.12–5.33 (m, 1.5H, =CH_2_, =CH), 5.49–5.58 (m, 0.5H, =CH), 5.71–5.85 (m, 0.5H, =CH), 6.13–6.24 (m, 0.5H, =CH), 6.29–6.37 (m, 0.5H, =CH), 6.56–6.70 (m, 0.5H, =CH); HRMS (ESI) *m*/*z* 279.1937 (calcd. for C_15_H_28_O_3_Na [M + Na]^+^, 279.1931).

*(3R,4S)-Methyl 3,4-dihydroxy-5-undecyl-tetrahydrofuran-2-acetate* (**7**). *n*-Butyl lithium (5.5 mL, 1.6 M) was added dropwise to a solution of triethyl phosphonoacetate (1.93 g, 8.6 mmol) in 70 mL of THF at −30 °C. After 0.5 h, a solution of compound **12a** (1.89 g, 6.0 mmol) in 5 mL of THF was added to the mixture, which was then allowed to warm to room temperature. The reaction mixture was stirred overnight and then filtered. The filtrate was dried over Na_2_SO_4_, filtered and concentrated. The residue was purified by column chromatography on silica gel (ethyl acetate/petroleum ether = 1:20) to give compound **15a** as a colorless oil (1.02 g, 44%). Compound **15a** (1.0 g, 2.6 mmol) was added to a solution of concentrated HCl (20 mL) in water/methanol (150 mL, *v*/*v* = 1:9). The mixture was stirred for 3 h at room temperature and then neutralized with NaOH solution. The resulting mixture was concentrated to remove methanol. The residue was diluted with EtOAc and washed with water. The organic phase was dried over Na_2_SO_4_, filtered and concentrated. The residue was purified by column chromatography on silica gel (ethyl acetate/petroleum ether = 1:3) to give compound **7** as a colorless oil (0.51 g, 69%). ^1^H-NMR (400 MHz, CDCl_3_) δ: 0.86 (t, *J* = 6.8 Hz, 3H, CH_3_), 1.24–1.72 (m, 20H, 10CH_2_), 2.58–2.86 (m, 2H, CH_2_), 3.60–3.90 (m containing s, 5H, CH, OCH_3_), 3.96–4.02 (m, 1H, CH), 4.79–4.98 (m, 1H, CH); HRMS (ESI) *m*/*z* 353.2287 (calcd. for C_18_H_34_O_5_Na [M + Na]^+^, 353.2298).

*(3R,4S)-Methyl 5-decyl-3,4-dihydroxytetrahydrofuran-2-acetate* (**8**). *n*-Butyl lithium (4.1 mL, 1.6 M) was added dropwise to a solution of triethyl phosphonoacetate (1.45 g, 6.45 mmol) in 70 mL of THF at −30 °C. After 0.5 h, a solution of compound **12b** (1.3 g, 4.3 mmol) in 5 mL of THF was added to the mixture, which was then allowed to warm to room temperature. The reaction mixture was stirred overnight and then filtered. The filtrate was dried over Na_2_SO_4_, filtered and concentrated. The residue was purified by column chromatography on silica gel (ethyl acetate/petroleum ether = 1:20) to give compound **15b** as a colorless oil (0.76 g, 48%). Compound **15b** (0.74 g, 2 mmol) was added to a solution of concentrated HCl (8 mL) in water/methanol (50 mL, *v*/*v* = 1:9). The mixture was stirred for 3 h at room temperature and then neutralized with NaOH solution. The resulting mixture was concentrated to remove methanol. The residue was diluted with EtOAc and washed with water. The organic phase was dried over Na_2_SO_4_, filtered and concentrated. The residue was purified by column chromatography on silica gel (ethyl acetate/petroleum ether = 1:3) to give compound **8** as a colorless oil (0.47 g, 75%). ^1^H-NMR (400 MHz, CDCl_3_) δ: 0.86 (t, *J* = 0.90 Hz, 3H, CH_3_), 1.23–1.55 (m, 18H, 9CH_2_), 2.61 (dd, *J* = 16.2, 7.4 Hz, 1H, CH_2_), 2.75 (dd, *J* = 16.2, 5.9 Hz, 1H, CH_2_), 3.78 (s, 3H, OCH_3_), 3.71–3.75 (m, 1H, CH), 3.78–3.85 (m, 2H, 2CH), 3.95–4.00 (m, 1H, CH); ^13^C NMR (100 MHz, CDCl_3_) δ: 14.1 (CH_3_), 22.6 (CH_2_), 25.5 (CH_2_), 29.3 (CH_2_), 29.5 (2CH_2_), 29.6 (3CH_2_), 31.9 (CH_2_), 38.3 (CH_2_), 52.0 (OCH_3_), 74.8 (CH), 74.9 (CH), 78.4 (CH), 84.3 (CH),172.5 (C=O); HRMS (ESI) *m*/*z* 339.2144 (calcd. for C_17_H_32_O_5_Na [M + Na]^+^, 339.2142).

*(3R,4S)-Methyl 3,4-dihydroxy-5-octyltetrahydrofuran-2-acetate* (**9**). *n*-Butyl lithium (4.3 mL, 1.6 M) was added dropwise to a solution of triethyl phosphonoacetate (1.28 g, 5.7 mmol) in 80 mL of THF at −10 °C. After 0.5 h, a solution of compound **12c** (1.04 g, 3.8 mmol) in 5 mL of THF was added to the mixture, which was then allowed to warm to 20 °C. The reaction was continued for another 6 h. The resulting mixture was filtered. The filtrate was dried over Na_2_SO_4_, filtered and concentrated. The residue was purified by column chromatography on silica gel (ethyl acetate/petroleum ether = 1:20) to give compound **15c** as a colorless oil (0.53 g, 41%). Compound **15c** (0.52 g, 1.5 mmol) was added to a solution of concentrated HCl (6 mL) in water/methanol (40 mL, *v*/*v* = 1:9). The mixture was stirred for 3 h at room temperature and then neutralized with NaOH solution. The resulting mixture was concentrated to remove methanol. The residue was diluted with EtOAc and washed with water. The organic phase was dried over Na_2_SO_4_, filtered and concentrated. The residue was purified by column chromatography on silica gel (ethyl acetate/petroleum ether = 1:3) to give compound **9** as a colorless oil (0.32 g, 74%). ^1^H-NMR (400 MHz, CDCl_3_) δ: 0.85 (t, *J* = 7.2 Hz, 3H, CH_3_), 1.23–1.73 (m, 14H, 7 CH_2_), 2.58–2.85 (m, 2H, CH_2_), 3.57–4.01 (m containing s, 6H, CH_3_, 3CH), 4.55–4.99 (m, 1H, CH); HRMS (ESI) *m*/*z* 289.2007 (calcd. for C_15_H_29_O_5_ [M + H]^+^, 289.2010).

### 3.3. Antifungal Biology Assay

#### 3.3.1. Strain

The *P. digitatum*, *P. italicum* and *A. niger* strain was obtained from the College of Horticulture and Forestry Sciences, Huazhong Agricultural University. The strain was incubated with potato dextrose agar (PDA). The *P. italicum* conidial suspension was prepared by diluting with 0.1% Tween-80 sterile water. After three days incubation, the concentration was adjusted to 1 × 10^6^ conidia mL^−1^ by diluting with sterile distilled water using a hemocytometer.

#### 3.3.2. *In Vitro* Antifungal Activity [29]

The mycelium was inoculated by a hole punch uniformly in 70 mm sterile plastic Petri dishes. Afte a pre-study, the tested compounds were diluted with PDA to 15, 25, 50, 75, 150 ppm for *P. digitatum* and 50, 100, 200, 300, 400 ppm for *P. italicum* and *A. niger* detection. Control plates contained culture medium without tested compounds. All the Petri dishes were incubated in biochemical incubator at 28 °C. Colony diameters (D) were measured after fourdays for *P. digitatum*, seven days for *P. italicum* and *A. niger*. Three measurements were taken on each plates and in triplicate for each concentration. The mean value was used for statistical analysis. Inhibitory rate% = [(D_control_ − D_test_)/D_control_] × 100%.

Plot was set with inhibitory rate as Y axial and concentration as X axial. IC_50_, the half maximal concentration to inhibit 50% of fungal activity, was calculated from the plot.

#### 3.3.3. *In Vivo* Antifungal Activity

*Citrus reticulate* used for experiments were collected from orchards in Wuhan, China. After being stored for one week at room temperature, tested fruits were selected for uniformity of size and maturity without any physical injury or infection. All fruits were washed with tap water and dried with tissue carefully before use. A uniform wound (3 mm deep and 4 mm wide) was made at the equator of each orange with a sterile needle. Then, 20 min later, 20 μL *P. italicum* conidial suspension was injected into each wound. After 2 h, 20 μL 300 ppm oxylipin mimics were inoculated and 20 μL sterile water for control. The tested fruits were stored in artificial climatic box at 28 °C with the relative humidity of 90%. The disease incidence and lesion diameters were recorded for 10 days. Analysis of 10 fruits, in triplicate for each compound was done.

The disease index for fruit was observed by assessing the extent of total disease symptoms on each fruit surface using the following scale:

0: lesion diameter = 0 mm (no visible disease symptoms);
1: 1 mm ≤ lesion diameter ≤ 10 mm;
2: 10 mm < lesion diameter ≤ 20 mm;
3: 20 mm < lesion diameter ≤ 30 mm;
4: lesion diameter > 30 mm.

The disease index was calculated using the formula [30]: ∑(disease scale × number of fruit in each class)/(number of total fruit × highest disease scale) × 100. Lesion diameter was measured by a vernier caliper using the cross method, and the unit was milimeter.

### 3.4. Statistical Analysis

The analysis was carried out using Statistical Package of Social Sciences (SPSS 17.0, SPSS Inc. Chicago, IL, USA).

## 4. Conclusions

In conclusion, a series of oxylipin mimics were designed and synthesized. The *in vitro* bioassay indicated that all these oxylipin mimics showed significant inhibitory effect to the growth of *P. digitatum*, *P. italicum* and *A. niger*. The *in vivo* experiment on citrus-inoculated *P. italicum*. demonstrated that the most effective compound could reduce the disease severity to 60%. However, the mechanism for the antifungal activity of these oxylipin mimics needs further investigation.

## Supplementary Materials

Supplementary materials can be accessed at: http://www.mdpi.com/1420-3049/21/2/254/s1.
