# Supplementary material for: Synthesis of Oxylipin Mimics and Their Antifungal Activity against the Citrus Postharvest Pathogens
Source: Molecules. 2016 Feb 22;21(2):254. doi: 10.3390/molecules21020254 (PMC6273781; doi:10.3390/molecules21020254)
Supplement: Supplementary file 1 [file molecules-21-00254-s001.pdf]

Supplementary Materials: Synthesis of Oxylipin Mimics and Their Antifungal Activity against the Citrus Postharvest Pathogens

Jimei Ma, Yupeng Li, Hangwei Chen, Zhen Zeng, Zi-Long Li and Hong Jiang*

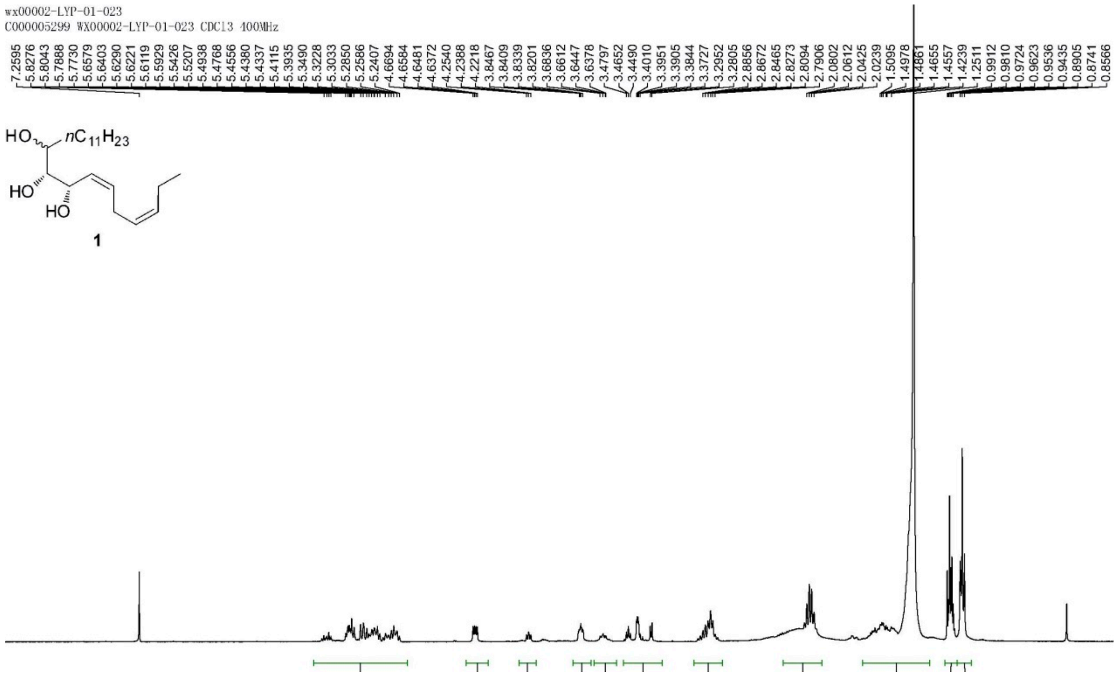

Figure 1. ^{1}H-NMR spectrum of compound **1**.

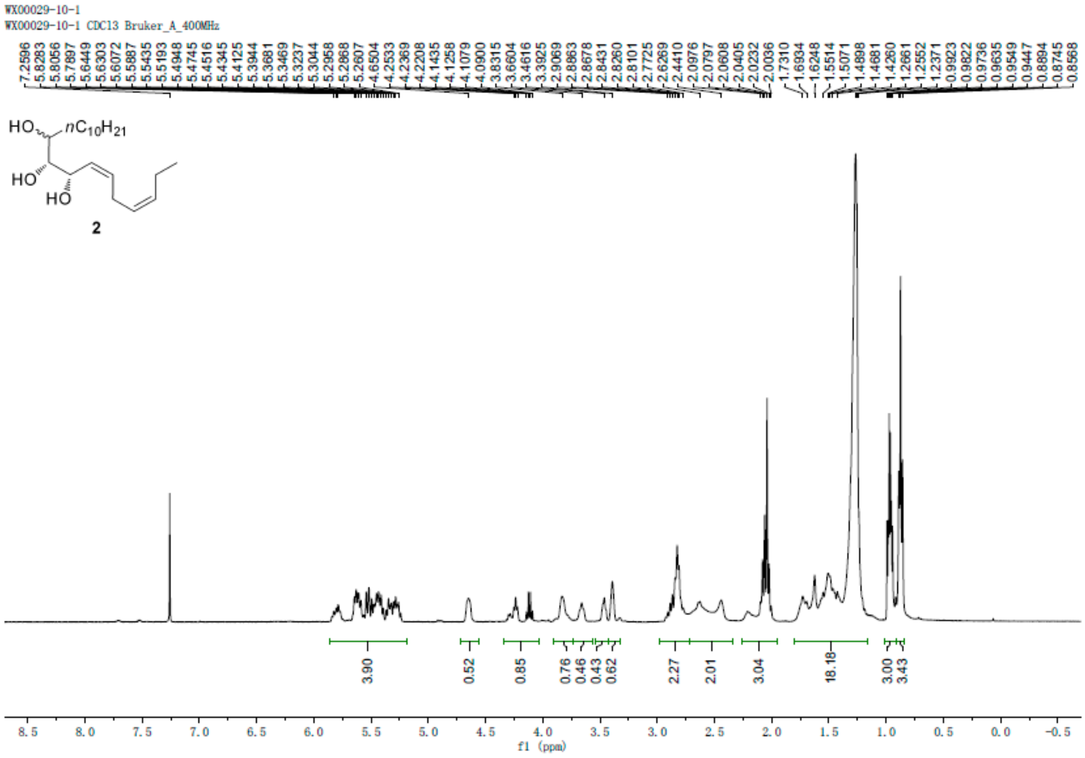

Figure 2. ^{1}H-NMR spectrum of compound **2**.

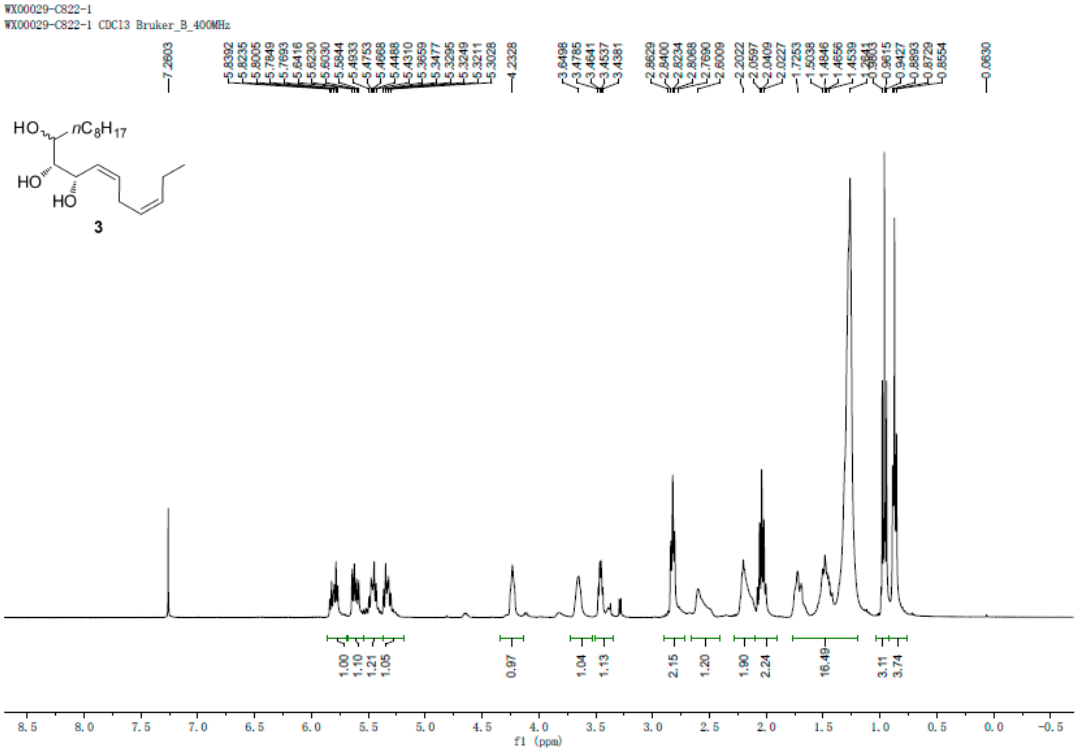

Figure 3. ^{1}H-NMR spectrum of compound **3**.

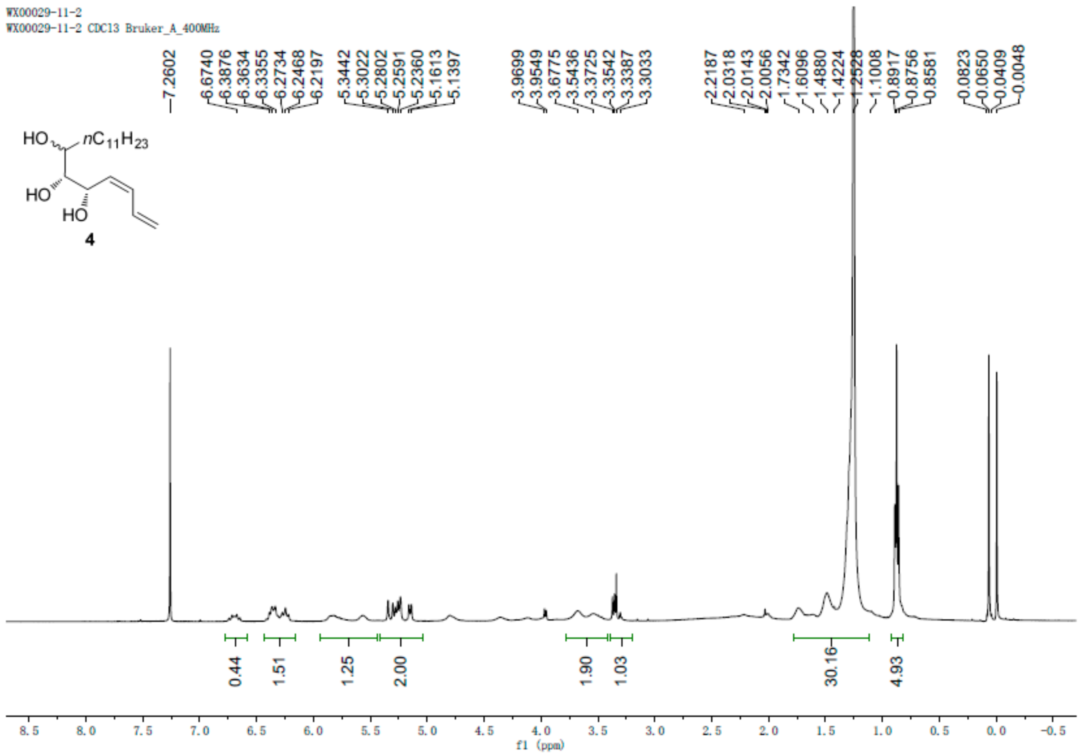

Figure 4. ^{1}H-NMR spectrum of compound **4**.

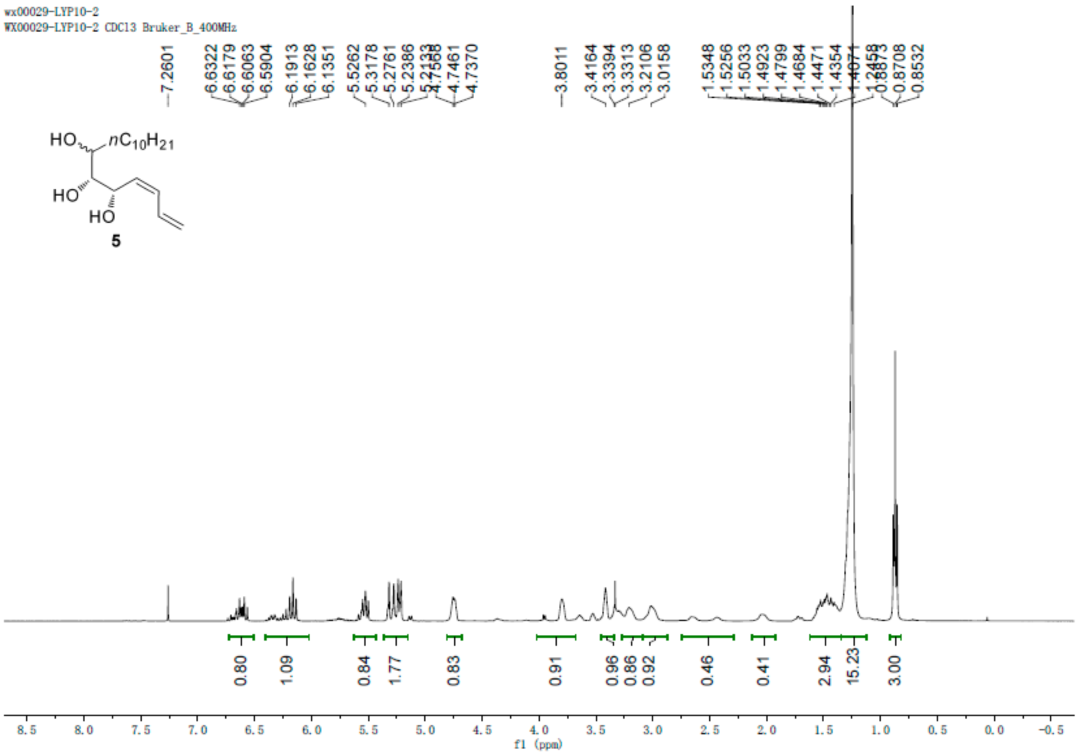

Figure 5. ^{1}H-NMR spectrum of compound **5**.

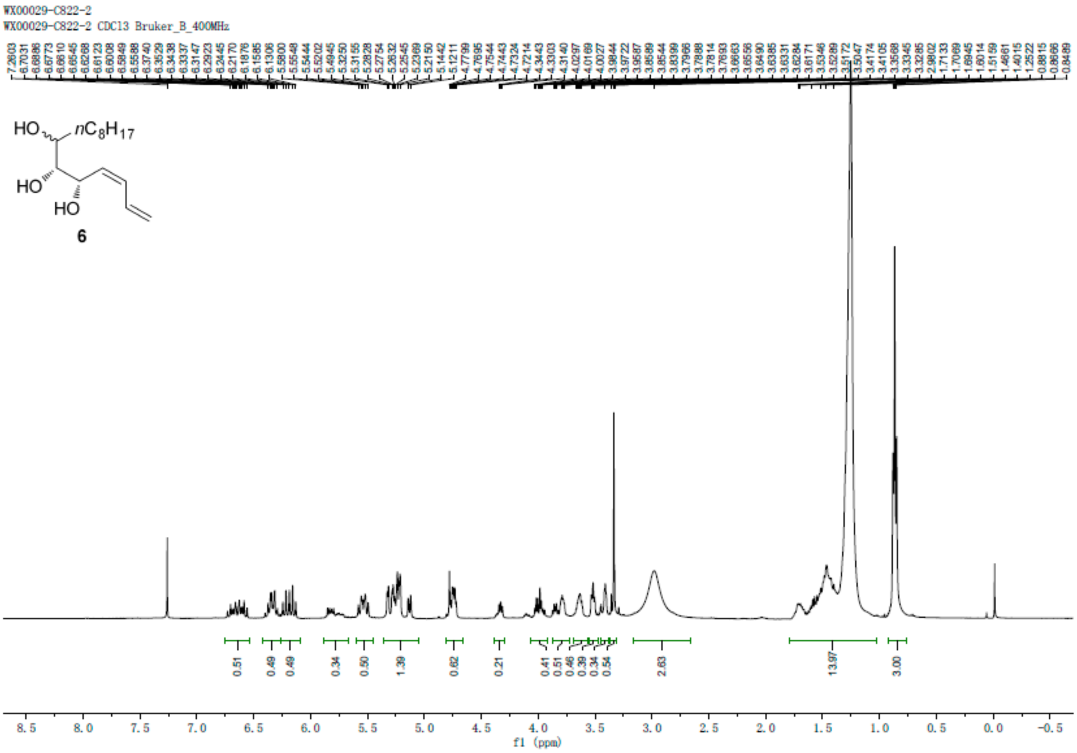

Figure 6. ^{1}H-NMR spectrum of compound **6**.

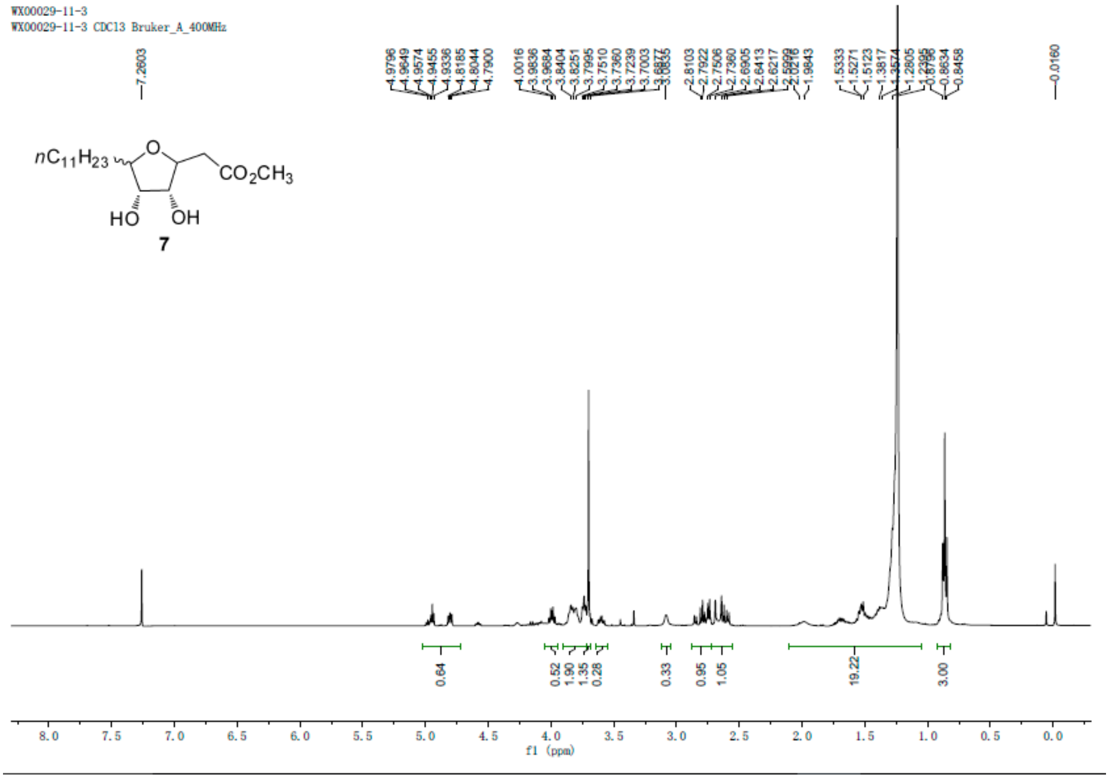

Figure 7. ^{1}H-NMR spectrum of compound **7**.

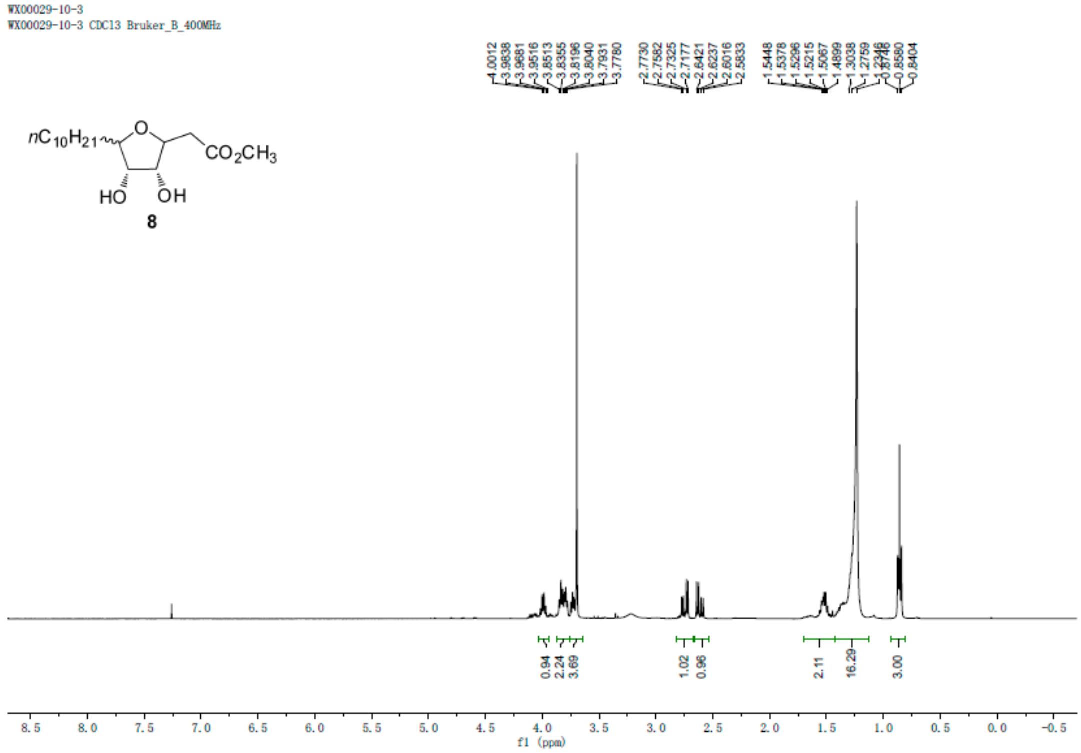

Figure 8. ^{1}H-NMR spectrum of compound **8**.

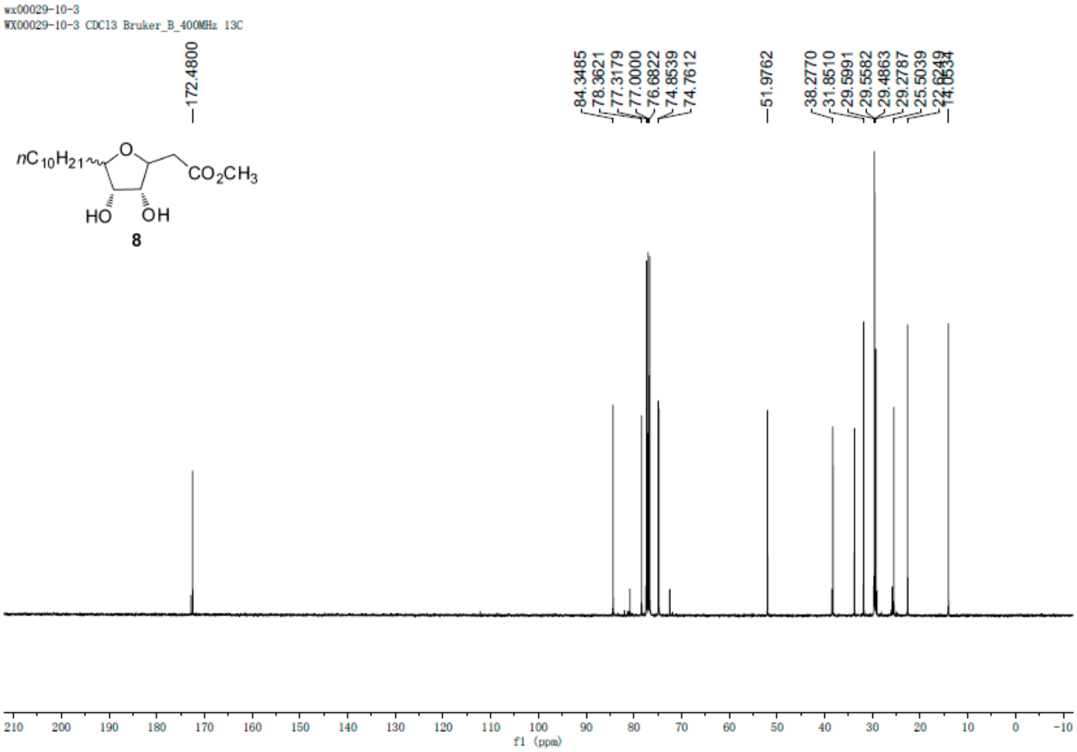

Figure 9. ^{13}C-NMR spectrum of compound **8**.

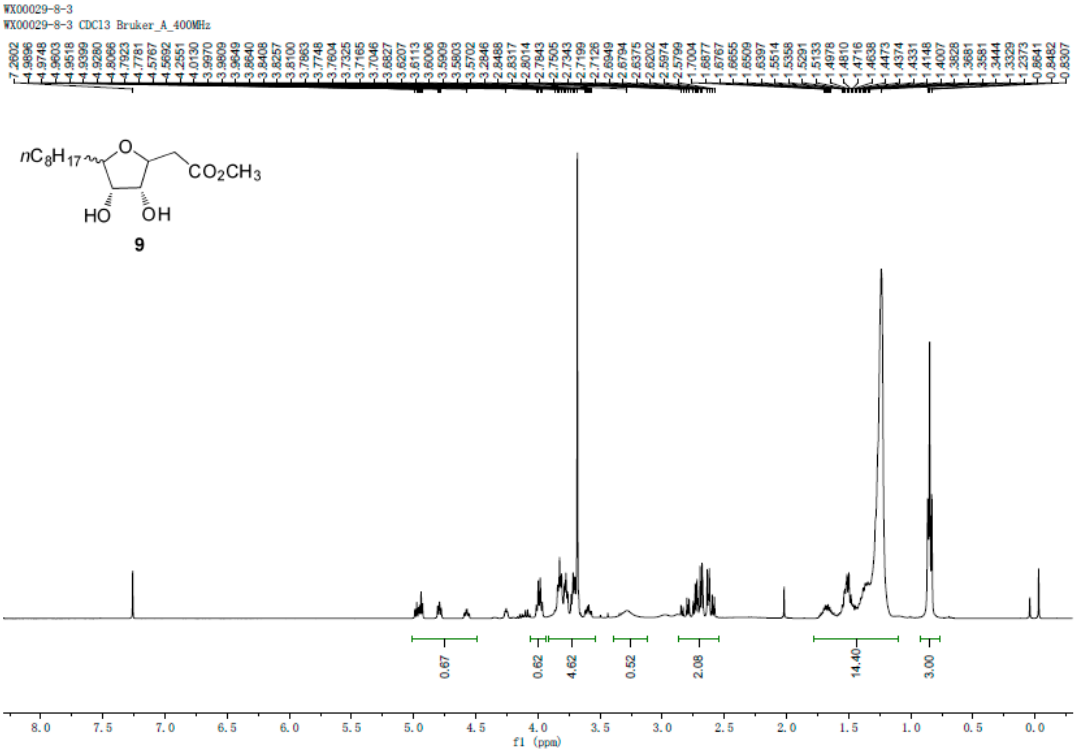

Figure 10. ^{1}H-NMR spectrum of compound **9**.

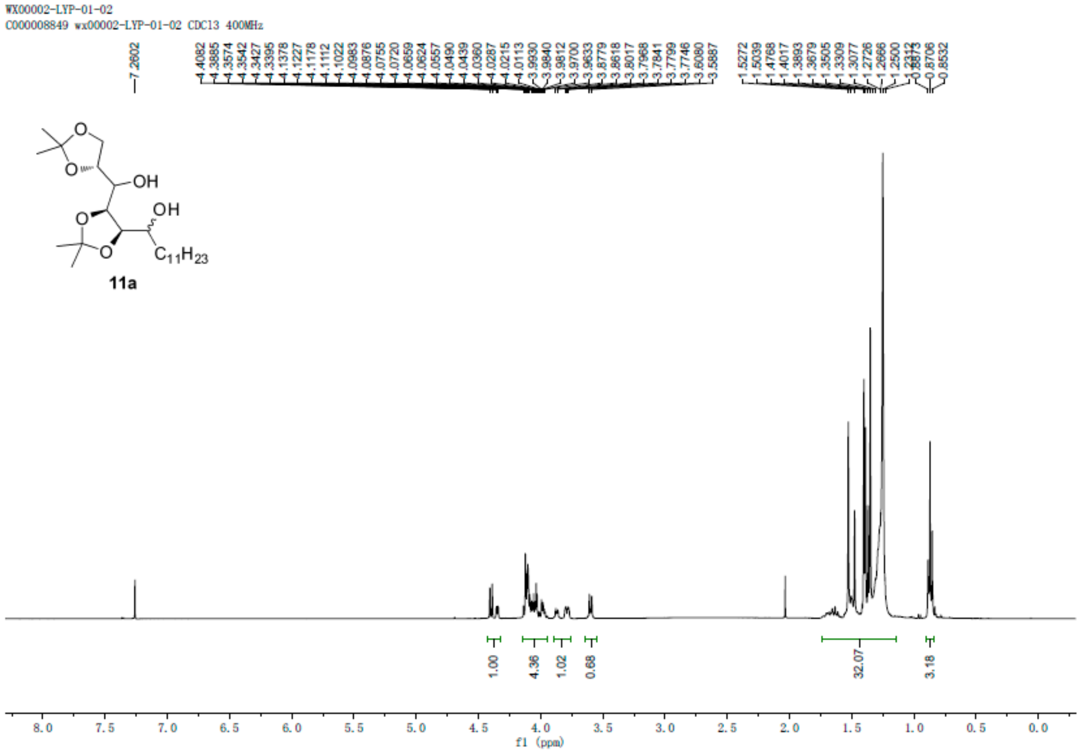

Figure 11. ^{1}H-NMR spectrum of compound **11a**.

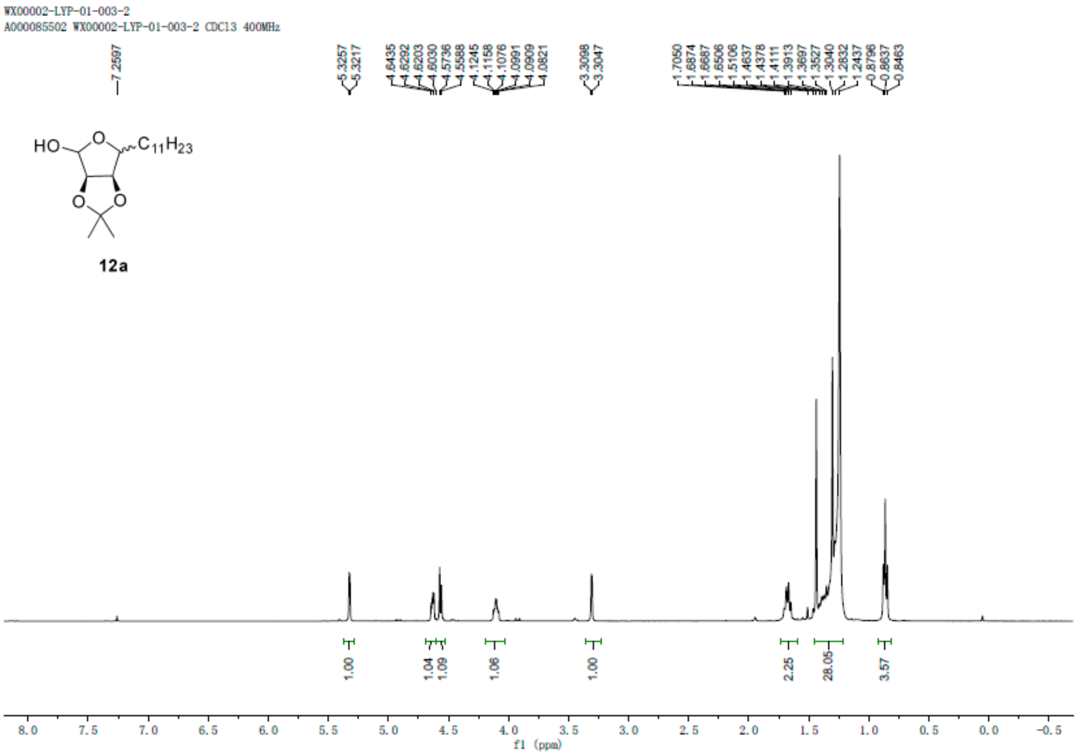

Figure 12. ^{1}H-NMR spectrum of compound **12a**.

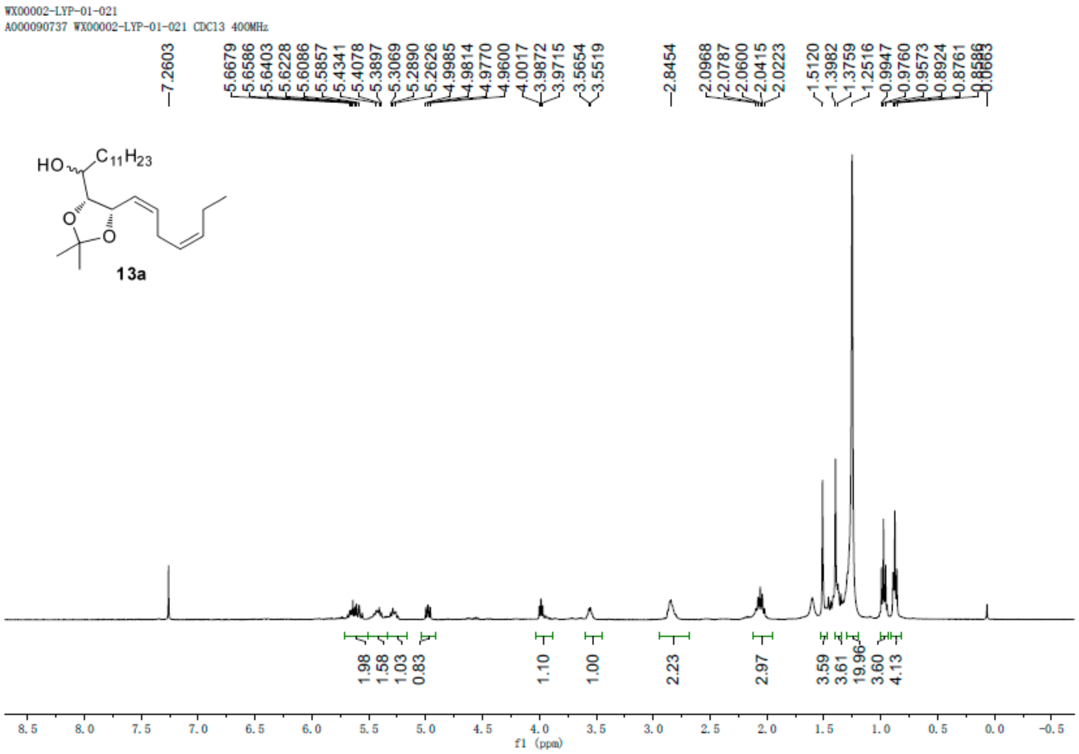

Figure 13. ^{1}H-NMR spectrum of compound **13a**.

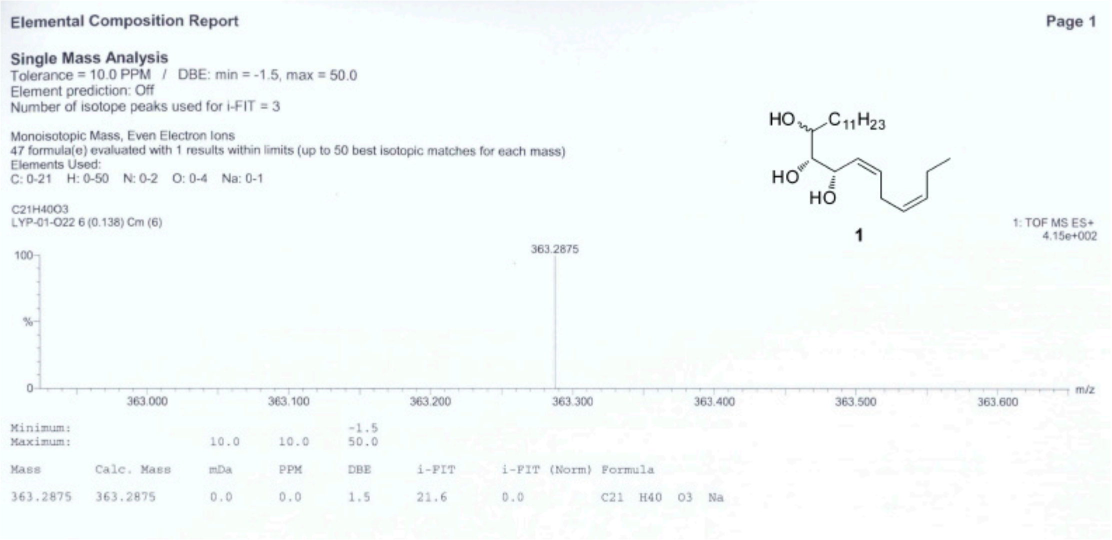

Figure 14. HRMS spectrum of compound **1**.

Acquisition Parameter

Source Type	ESI	Ion Polarity	Positive	Set Nebulizer	2.5 Bar
Focus	Not active			Set Dry Heater	200 °C
Scan Begin	50 m/z	Set Capillary	4500 V	Set Dry Gas	6.0 l/min
Scan End	3000 m/z	Set End Plate Offset	-500 V	Set Divert Valve	Waste

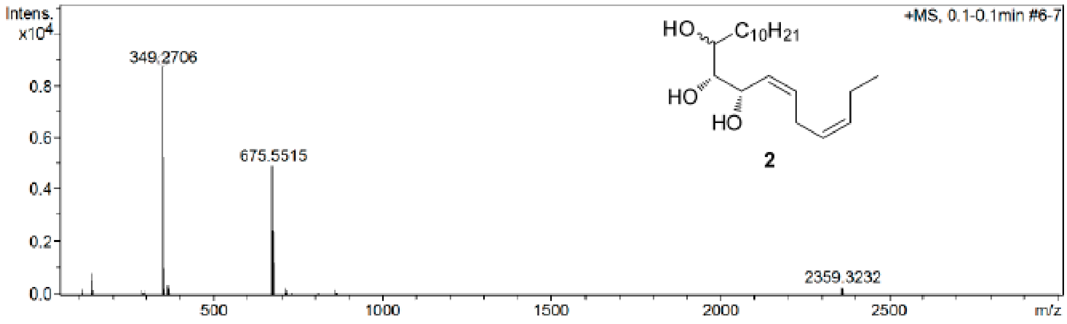

Meas. m/z	#	Ion Formula	m/z	err [ppm]	mSigma	# mSigma	Score	rdb	e⁻ Conf	N-Rule
349.2706	1	C20H38NaO3	349.2713	2.2	5.9	1	100.00	1.5	even	ok

Figure 15. HRMS spectrum of compound **2**.

Acquisition Parameter

Source Type	ESI	Ion Polarity	Positive	Set Nebulizer	2.5 Bar
Focus	Not active			Set Dry Heater	200 °C
Scan Begin	50 m/z	Set Capillary	4500 V	Set Dry Gas	6.0 l/min
Scan End	3000 m/z	Set End Plate Offset	-500 V	Set Divert Valve	Waste

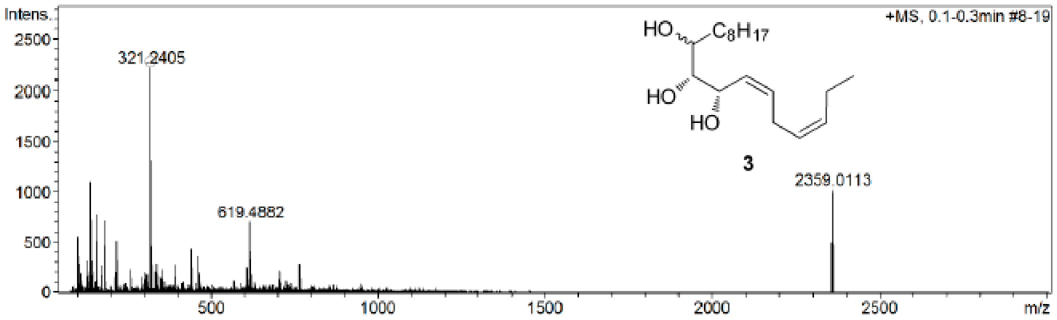

Meas. m/z	#	Ion Formula	m/z	err [ppm]	mSigma	# mSigma	Score	rdb	e⁻ Conf	N-Rule
321.2405	1	C18H34NaO3	321.2400	-1.4	16.6	1	100.00	1.5	even	ok

Figure 16. HRMS spectrum of compound **3**.

Acquisition Parameter

Source Type	ESI	Ion Polarity	Positive	Set Nebulizer	2.5 Bar
Focus	Not active			Set Dry Heater	200 °C
Scan Begin	50 m/z	Set Capillary	4500 V	Set Dry Gas	6.0 l/min
Scan End	3000 m/z	Set End Plate Offset	-500 V	Set Divert Valve	Waste

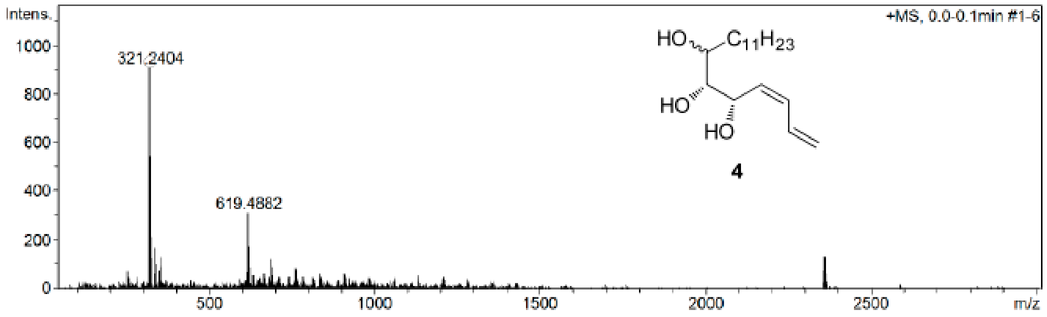

Meas. m/z	#	Ion Formula	m/z	err [ppm]	mSigma	# mSigma	Score	rdb	e⁻ Conf	N-Rule
321.2404	1	C18H34NaO3	321.2400	-1.3	24.8	1	100.00	1.5	even	ok

Figure 17. HRMS spectrum of compound **4**.

Acquisition Parameter

Source Type	ESI	Ion Polarity	Positive	Set Nebulizer	2.5 Bar
Focus	Not active			Set Dry Heater	200 °C
Scan Begin	50 m/z	Set Capillary	4500 V	Set Dry Gas	6.0 l/min
Scan End	3000 m/z	Set End Plate Offset	-500 V	Set Divert Valve	Waste

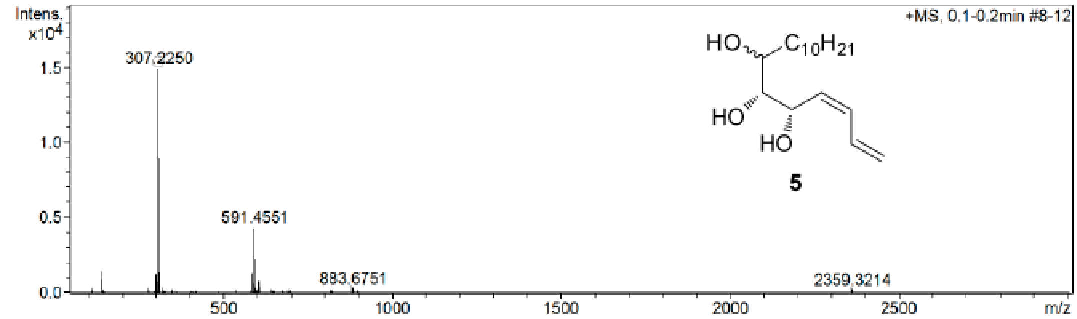

Meas. m/z	#	Ion Formula	m/z	err [ppm]	mSigma	# mSigma	Score	rdb	e⁻ Conf	N-Rule
307.2250	1	C17H32NaO3	307.2244	-2.0	2.0	1	100.00	1.5	even	ok

Figure 18. HRMS spectrum of compound **5**.

Acquisition Parameter

Source Type	ESI	Ion Polarity	Positive	Set Nebulizer	2.5 Bar
Focus	Not active			Set Dry Heater	200 °C
Scan Begin	50 m/z	Set Capillary	4500 V	Set Dry Gas	6.0 l/min
Scan End	3000 m/z	Set End Plate Offset	-500 V	Set Divert Valve	Waste

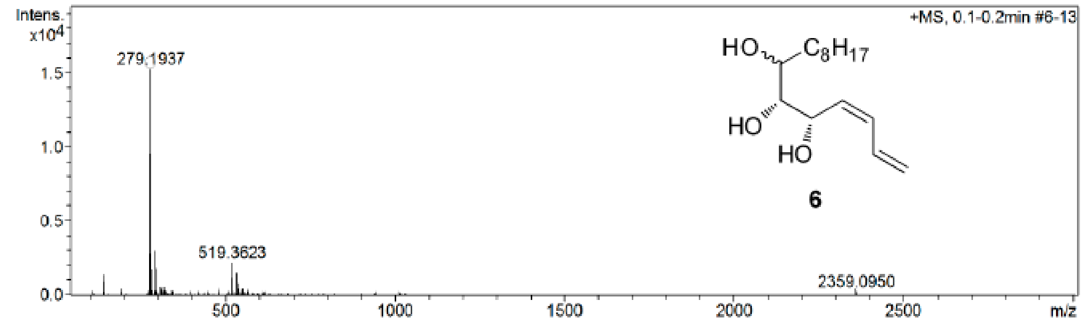

Meas. m/z	#	Ion Formula	m/z	err [ppm]	mSigma	# mSigma	Score	rdb	e⁻ Conf	N-Rule
279.1937	1	C15H28NaO3	279.1931	-2.4	14.4	1	100.00	1.5	even	ok

Figure 19. HRMS spectrum of compound **6**.

Acquisition Parameter

Source Type	ESI	Ion Polarity	Positive	Set Nebulizer	2.5 Bar
Focus	Not active			Set Dry Heater	200 °C
Scan Begin	50 m/z	Set Capillary	4500 V	Set Dry Gas	6.0 l/min
Scan End	3000 m/z	Set End Plate Offset	-500 V	Set Divert Valve	Waste

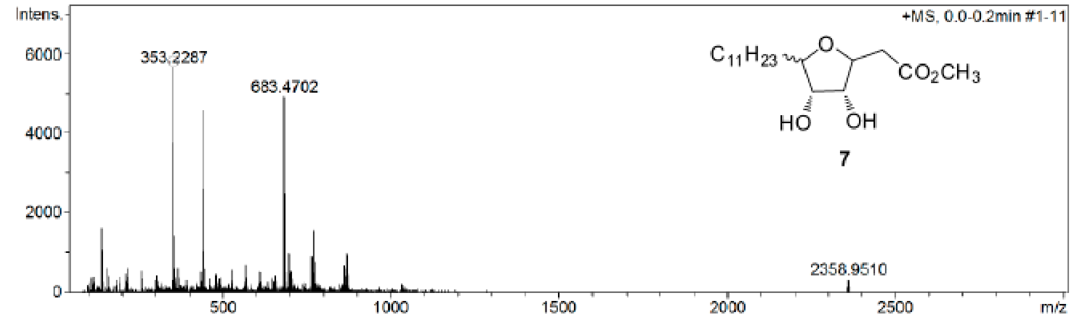

Meas. m/z	#	Ion Formula	m/z	err [ppm]	mSigma	# mSigma	Score	rdb	e⁻ Conf	N-Rule
353.2287	1	C18H34NaO5	353.2298	3.2	22.4	1	100.00	1.5	even	ok

Figure 20. HRMS spectrum of compound **7**.

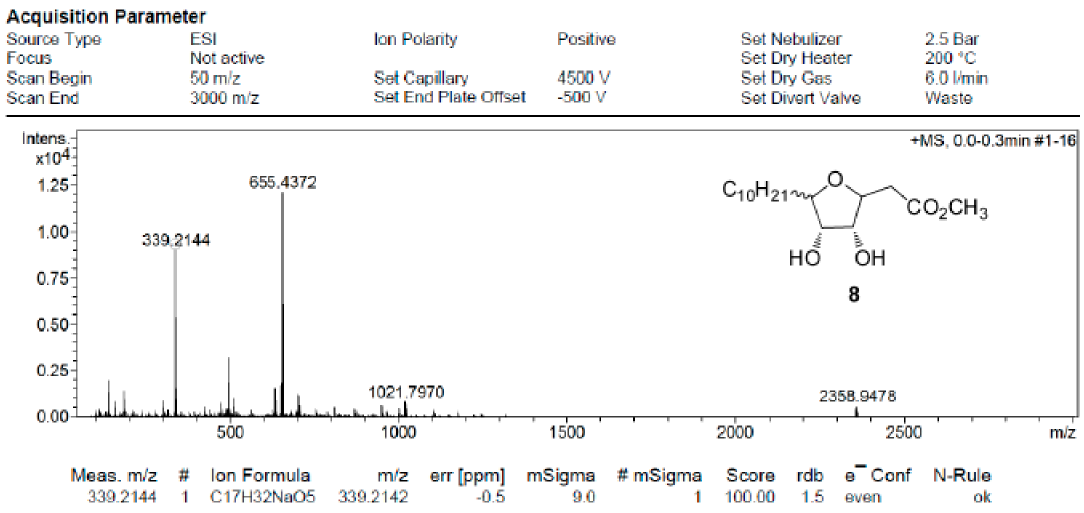

Figure 21. HRMS spectrum of compound **8**.

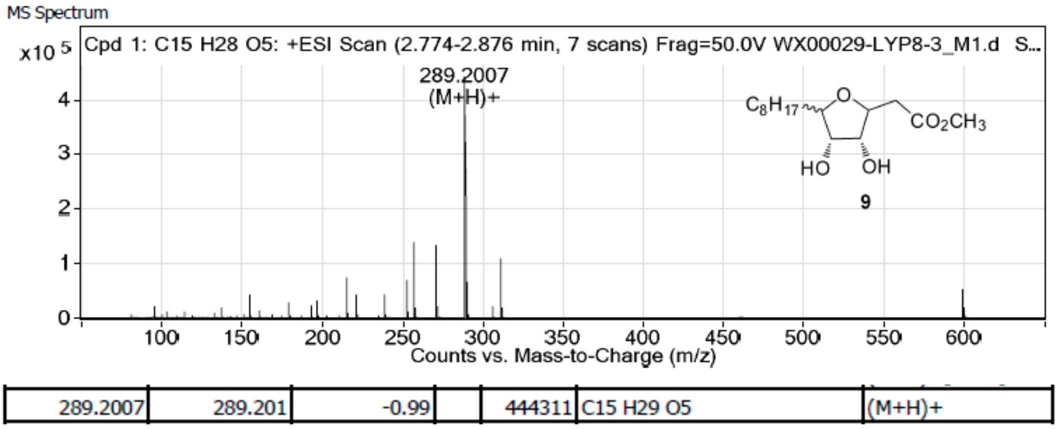

Figure 22. HRMS spectrum of compound **9**.

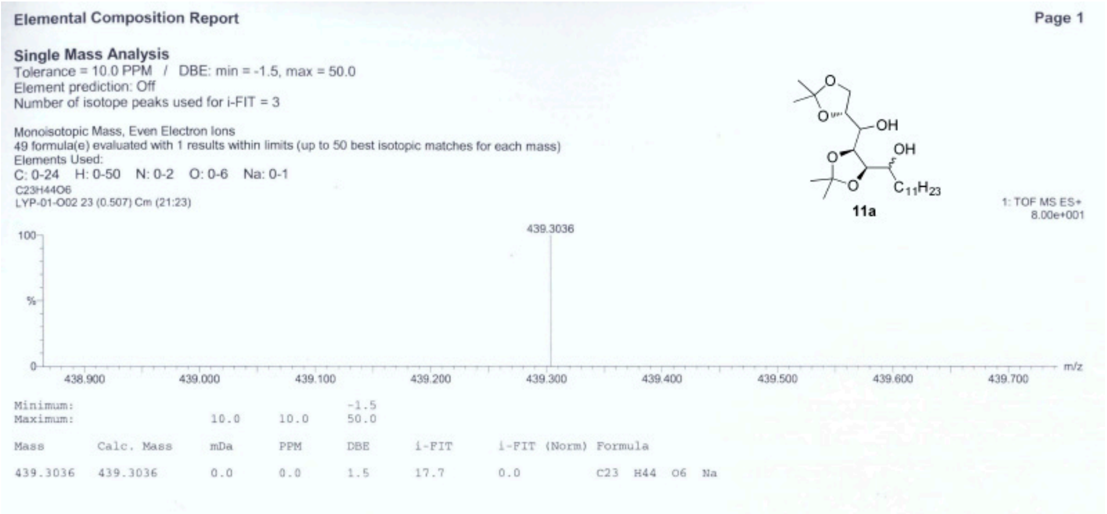

Figure 23. HRMS spectrum of compound **11a**.

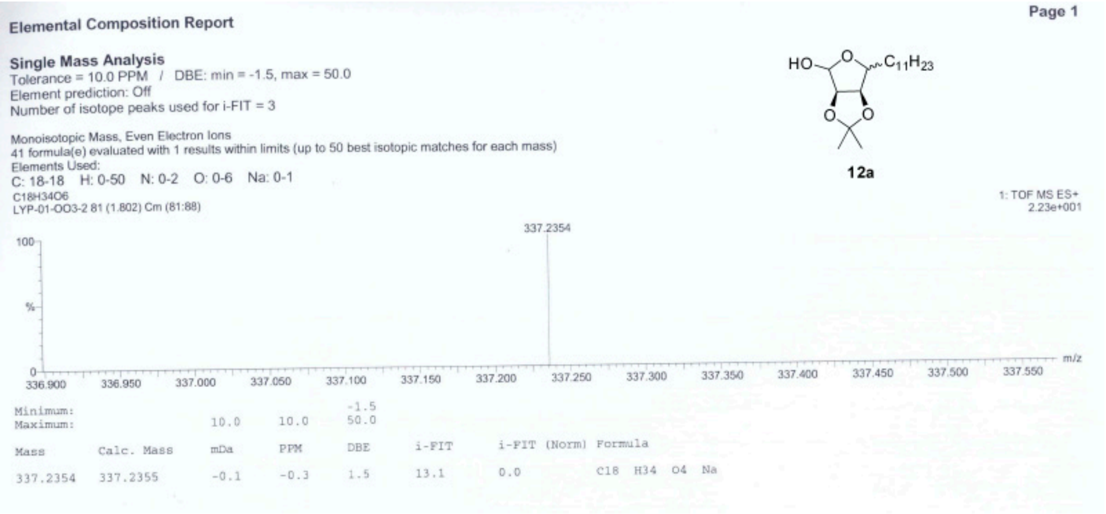

Figure 24. HRMS spectrum of compound **12a**.

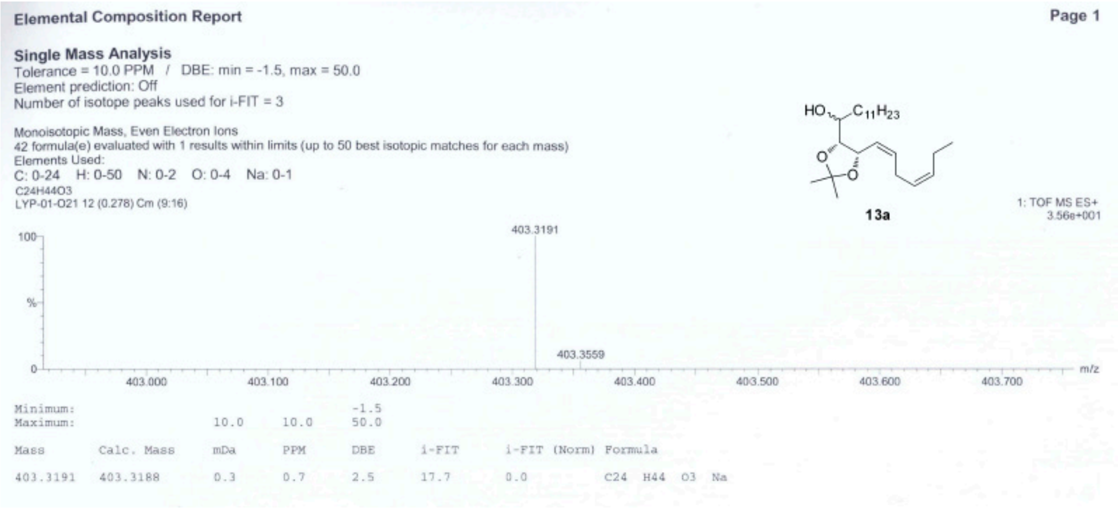

Figure 25. HRMS spectrum of compound **13a**.
